# Cryopreservation and validation of differentiated PDGFRα-positive cells for long term usage in experimentation

**DOI:** 10.1186/s13104-023-06549-y

**Published:** 2023-10-19

**Authors:** Ankita Srivastava, Reeja Rajan, Sallam Hasan Abdallah, Amir Ali Khan, Bashair M. Mussa

**Affiliations:** 1 College of Medicine, Research Institute of Medical and Health Sciences, University of Sharjah, P.O. Box: 27272, Sharjah, United Arab Emirates; 2 Research Institute of Sciences and Engineering, University of Sharjah, P.O. Box: 27272, Sharjah, United Arab Emirates; 3 The Department of Biomedical and Clinical Sciences, Linkoping University, Linkoping, 58183 Sweden; 4 Department of Applied Biology, College of Science, University of Sharjah, P.O. Box: 27272, Sharjah, United Arab Emirates; 5 BioGrad, 61 Stephenson Way, Liverpool, L13 1HN UK; 6 Basic Medical Science Department, College of Medicine, University of Sharjah, P.O. Box: 27272, Sharjah, United Arab Emirates

**Keywords:** iMSCs, Stem cell differentiation, PDGFRα-positive cells, qRT-PCR, Flow cytometry, Western blotting

## Abstract

**Objective:**

Differentiation of immortalized Mesenchymal Stromal Cells (iMSCs) into PDGFRα-positive cells under controlled growth conditions has several vital implications in functional studies concerned with the pathogenesis of Diabetic Gastroparesis (DGP). A study published previously by our research group demonstrated the importance of these cells as a novel, in-vitro model for investigating the functional role of neuronal nitric oxide synthase. The currently available methods require fresh differentiation of PDGFRα-positive cells for each round of experimentation. This leads to longer delays, higher usage of reagents, and inconsistency in reproducibility of experiments frequently. We thus aimed to establish through validation that cryopreserving and maintaining the iMSC-derived PDGFRα-positive cells for functional investigations help us to overcome these challenges.

**Results:**

We demonstrated for the first time that the differentiated PDGFRα-positive cells from iMSCs can be cryopreserved and thawed to be used as per the experimental requirements with prolonged preservation of their characteristics. We assessed the viability of differentiated PDGFRα-positive cells pre- and post-freezing with the subsequent validation of their functional features using flow cytometry, qRT-PCR, and western blotting. We have been successful in demonstrating for the first time that the cryopreservation of previously differentiated PDGFRα-positive cells can be used as a feasible and cost-effective model for experimental reproducibility in functional studies of Diabetes Gastroparesis.

**Graphical Abstract:**

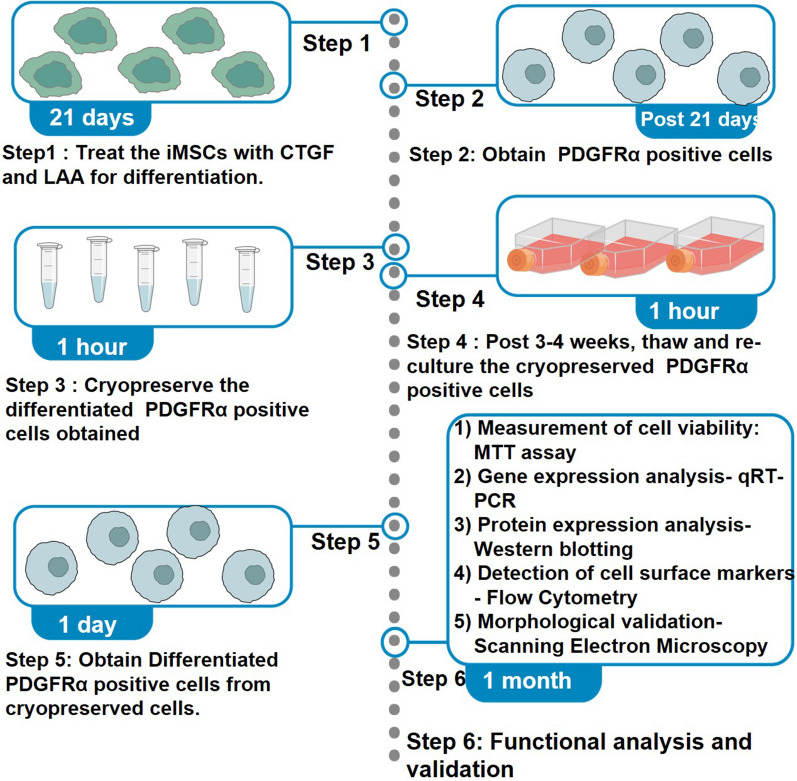

**Supplementary Information:**

The online version contains supplementary material available at 10.1186/s13104-023-06549-y.

## Introduction

**Fig. 1:**
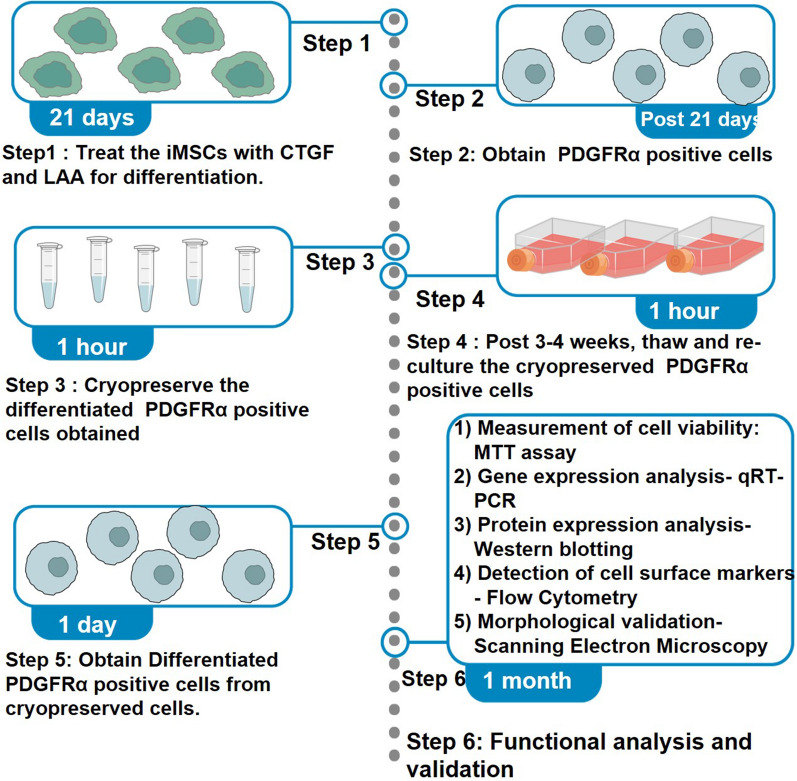
Cryopreservation, thawing and maintenance of differentiated PDGFRα-positive cells. iMSCs were treated with connective tissue growth factor (CTGF) and l-ascorbic acid (LAA) for a period of 21 days to allow their differentiation into PDGFRα-positive cells. Post day 21, the cells were cryopreserved for 3–4 weeks as a new approach, then thawed and sub-cultured. The survival of the cells was assessed using MTT assay and functional validation was conducted using flow cytometry for detection of cell surface markers, qRT-PCR for gene expression analysis and western blotting for protein expression analysis

A study published by our research group [1] has established that telomerase transformed immortalized Mesenchymal Stromal Cells (iMSCs) can be successfully differentiated into PDGFRα-positive cells using connective tissue growth factor (CTGF) and l-ascorbic acid (LAA) [1, 2]. The iMSC-derived PDGFRα-positive cells thus obtained have been successfully used as an in-vitro model to conduct functional studies associated with Diabetic Gastroparesis [1, 3]. We and others have used this protocol to differentiate iMSCs into PDGFRα-positive cells; however, one of the limitations of the protocol was the waiting time of 21 days for each round of differentiation. Therefore, we worked towards the idea of using cryopreservation as a method for maintaining these cells post day 21 of differentiation and the possible usage of these cells post thawing preferably after 3–4 weeks to render them useful in future experiments. This method would help investigators have ready to use cryopreserved differentiated PDGFRα-positive cells instead of having repeated rounds of 21-day differentiation to execute their experimentation. Figure 1 illustrates an overview of the differentiation, maintenance and cryopreservation process.

We have also described the details of the various steps used in validating the PDGFRα-positive cells during long-term storage in liquid nitrogen and their subsequent thawing and compared the results to a positive control (primary fibroblasts [F180]) and negative control (undifferentiated iMSCs). These steps involve analyzing cell survivability using MTT, detecting specific cell surface markers using flow cytometry, and analyzing gene and protein expression using qRT-PCR and western blotting, respectively. The preservation of morphology of the differentiated cells were validated using Scanning electron microscopy.

## Main text

### Experimental design and methodology

#### Cell culture and fibroblastic differentiation

(i) **Reagents and consumables required**
  - Negative control: telomerase transformed immortalized human bone marrow-derived mesenchymal stromal cells (iMSCs; RRID: CVCL_B5PE); Applied Biological Materials, Richmond, Canada; Catalogue No. abm T0529.
  - Positive control: primary human fibroblasts [F180]; University Hospital Sharjah, Sharjah, UAE.
  - 6 well plates; Jet Biofil, Guangzhou, China.
  - Minimum Essential Medium-alpha modification (MEM-α); Sigma Aldrich, St. Louis, MO, USA; Catalogue No. 32571-028.
  - Fetal Bovine Serum; Sigma Aldrich, St. Louis, MO, USA; Catalogue No. F7524.
  - l-Glutamine; Sigma Aldrich, St. Louis, MO, USA; Catalogue No. G3126-500G.
  - Penicillin–Streptomycin; Sigma Aldrich, St. Louis, MO, USA; Catalogue No. P4333-100ML.
  - Connective tissue growth factor (CTGF); Biovendor, Brno, Czech Republic; Ref Code: RD191035299R.
  - l-Ascorbic acid (LAA); Sigma Aldrich, St. Louis, MO, USA; Catalogue No. A92902.
  - T-25 and T-75 cell culture flasks; Sigma Aldrich, St. Louis, MO, USA.
  - Trypsin–EDTA solution; Sigma Aldrich, St. Louis, MO, USA; Catalogue No. T4299.
  - Dulbecco’s Phosphate Buffered Saline (PBS); Sigma Aldrich, St. Louis, MO, USA; Catalogue No. D8537
  - Dulbecco’s Modified Eagle Media (DMEM); Sigma Aldrich, St. Louis, MO, USA; Catalogue No. D5671.
  - Media recipes
    1. **Complete media: MEM-α and DMEM**
      To MEM-α (reagents #1.1d), 0.292 g/L of l-glutamine [reagents #3.1(i)f], 10% heat inactivated FBS [reagents #3.1(i)e] and 1% Penicillin–Streptomycin [reagents #3.1(i)g] were added. To DMEM [reagents #3.1(i)m], 10% heat inactivated FBS and 1% Penicillin–Streptomycin were added.
    2. **Fibroblastic differentiation medium**
      Complete MEM-α supplemented with 100 ng/L CTGF [reagents# 3.1(i)h] and 50 µg/mL LAA [reagents #3.1(i)i].
(ii) **Methodology**
  1. Culture telomerase transformed iMSCs [Reagents 3.1(i)a] in complete MEM-α (Media recipes n#1) in a humidified atmosphere of 5% CO2 at 37 °C in T-75 tissue culture flasks [Reagents #3.1(i)j].
  2. Harvest the cells at 80% confluency by washing the adherent cells with 5 mL of sterile PBS thrice and trypsinize them with the addition of 500ul of 0.25% trypsin–EDTA solution [Reagents #3.1(i)k] and incubating for 2 min at 37 °C. Terminate the tripsinization process by the addition of 5 mL of complete DMEM [Media Recipes n#1].
  3. Plate the iMSCs in 6-well plates [Reagents #3.1(i)1b] at a density of 250 × 10^3^ cells/well.
  4. Add fibroblastic differentiation medium when the cells reach 70–80% confluency (Media recipes n#2).
  5. Change the medium every day for 21 days.
  6. Use iMSCs [Reagents #3.1(i)a] cultured in MEM-α as negative control and primary human fibroblasts [F180] [Reagents #3.1(i)] cultured in DMEM as a positive control in the absence of CTGF and LAA.
  7. Subsequently, harvest the differentiated PDGFRα-positive cells for future studies on the 21st day for storage through cryopreservation.

#### Cryopreservation of the differentiated PDGFRα-positive cells

(i) **Reagents and consumables required**
  - 1.2 mL cryovials; Thermo Fisher Scientific, Waltham, MA, USA; Catalogue No. 5000-0012.
  - Trypsin–EDTA solution; Sigma Aldrich, St. Louis, MO, USA; Catalogue No. T4299.
  - Dulbecco’s Phosphate Buffered Saline (PBS); Sigma Aldrich, St. Louis, MO, USA; Catalogue No. D8537.
  - Complete MEM-α and DMEM media (Media recipes n #1).
  - Dimethyl sulfoxide (DMSO); Sigma Aldrich, St. Louis, MO, USA; Catalogue No. D8418.
(ii) **Methodology**
  1. On day 21, wash the differentiated PDGFRα-positive cells twice with PBS [Reagents #3.2(i)c] to remove the old media.
  2. Subsequently, add 500 µL of trypsin–EDTA [Reagents #3.2(i)b] and distribute over the entire surface area of the wells, ensuring complete coverage of the adherent cell layer and incubate at 37 °C for 2 min. Terminate the trypsinization process by the addition of complete DMEM [Media recipes n#1]
  3. Observe the cells under a microscope to confirm complete detachment, add fresh complete media (MEM-α, Media recipes n #1), disperse by gentle repeated pipetting to avoid clumping and achieve single-cell suspension. Similarly, for the controls, resuspend iMSCs in MEM-α and fibroblasts in DMEM (Media recipes n#1).
  4. Collect the cell suspension in 15 mL falcon tubes and centrifuge at 1500 rpm (g-101) at 24 °C for 5 min.
  5. CRITICAL STEP. Discard the supernatant and resuspend the pellets in 90% of fresh complete media (Media recipes n#1). To this, add 10% of cryoprotectant (DMSO; [Reagents #3.2(i)e] dropwise to reduce cell death.
  6. Distribute the above cell suspension into cryovials with approximately 1 × 10^6^ cells per vial [Reagents #3.2(i)a].
  7. Store the cryovials in a − 80 °C freezer for 24 h up to 1 week and then transfer them to a liquid nitrogen tank until future use.

#### Thawing and sub-culturing of the differentiated PDGFRα-positive cells

(i) **Methodology**
  1. Thaw the frozen cell suspension in cryovial stored at − 80 °C/liquid nitrogen in a 37 °C water bath for less than a minute with gentle swirling. Transfer it into a 15 mL sterile tube and add 4–6 mL of complete media (Media Recipes n#1).
  2. Centrifuge the above tube with cells at 1500 rpm for 5 min at 24 °C to get rid of DMSO.
  3. Discard the supernatant and resuspend the pellet in 4–6 mL of complete media. Transfer the cell suspension into a T-25 culture flask [Reagents #3.1(i) j]and incubate at 37 °C with 5% CO_2_.
  4. Over the following few days, observe the cells and change the media every two days until the cells are healthy and reach 60–80% confluency (up to 1 week).

Subculture the cells for one passage before using for further experimentation to achieve healthy differentiated cells. Change the medium (with CTGF and LAA) every 48 h.

#### Measuring cell viability using MTT

(i) **Reagents and consumables required**
  - 3-[4,5-Dimethylthiazol-2-yl]-2,5 diphenyltetrazolium bromide (MTT); Sigma-Aldrich, St.Louis, MO, USA; Catalogue No. CAS 298-93-1.
  - 96-well plates; Jet Biofil, Guangzhou, China.
  - Trypsin–EDTA solution; Sigma Aldrich, St. Louis, MO, USA; Catalogue No. T4299.
  - Dulbecco’s Phosphate Buffered Saline (PBS); Sigma Aldrich, St. Louis, MO, USA; Catalogue No. D8537.
  - Complete MEM-α and DMEM media (Media recipes n#1).
  - Dimethyl sulfoxide (DMSO); Sigma Aldrich, St. Louis, MO, USA; Catalogue No. D8418.
(ii) **Methodology**
  1. Use two sets of PDGFRα-positive cells for MTT analysis: pre-cryopreservation on day 21 (Group 1: before freezing) and post thawing after 3–4 weeks (Group 2: after freezing). Centrifuge the above tube with cells at 1500 rpm for 5 min at 24 °C to get rid of DMSO.
  2. Seed approximately 1 × 10^4^ PDGFRα-positive cells in triplicates in 96-well plates [Reagents #3.4(i)b] and maintain in 200 μL complete MEM-α medium (Media recipes n#1) at 37 °C with 5% CO_2_.
  3. After 72 h, discard the old culture medium and add 20 μL of MTT (5 mg/mL) [Reagents #3.4(i)a] dissolved in 100 μL of PBS [Reagents #4.1d] to each well.
  4. Discard the MTT + PBS solution carefully and add 100 μL of DMSO; [Reagents #3.4(i)f] per well.
  5. Record the absorbance at 570 nm on a microplate reader.

### Results

#### Investigation of viability of cells post cryopreservation using MTT assay

**Fig. 2:**
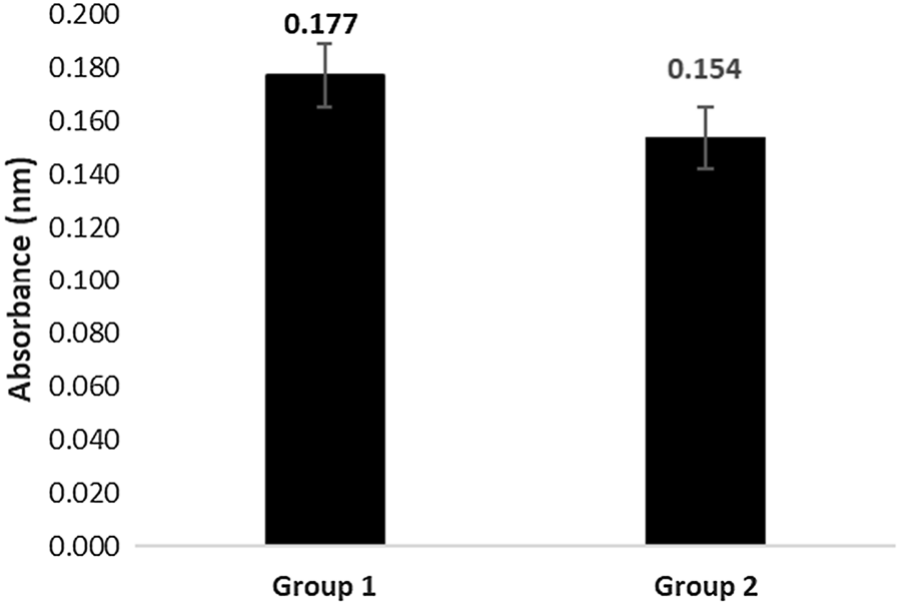
Comparing cell viability before and post cryopreservation. Using MTT cell viability assay, absorbance was recorded for assessing cell survival in group 1 (before freezing; 0.177 nm) and group 2 (after freezing; 0.154 nm). A minute difference of 2.3% was observed, which was not significant. Values represent average absorbance from three individual experiments

The comparison of cell viability before and after cryopreservation of cells was done using the MTT assay and only a minute difference of 2.3% was observed in the recovered percentage of cells post cryopreservation, the difference being non-significant proves that the viability of cells is not much affected due to the cryopreservation process (Fig. 2).

#### Confirming the presence of cell surface markers of differentiated PDGFRα-positive cells using flow cytometry analysis

The validation of the cryopreserved differentiated PDGFRα-positive cells was done by flowcytometry analysis. The expression of cell surface markers CD140α/PDGFRα, CD44, and CD34 were measured and analysed in iMSCs, fibroblasts, and PDGFRα-positive cells using flowcytometry. The details of the results can be obtained from published data [1] [10.3390/ijms22073514].

#### Gene expression analysis for validation of the cryopreserved and differentiated PDGFRα-positive cells

The validation of the cryopreserved differentiated PDGFRα-positive cells was done by measuring the Gene expression of Extracellular Matrix (ECM) Proteins (COL I, DEC, ELA, HAS3, and TIMP1) and Stem Cell Differentiation (SCD) markers (ALP, AGG, CD44, and FSP-1) in fibroblasts, iMSCs, and PDGFRα-positive cells. The details of the results from qRT PCR can be obtained from previously published data [1] [10.3390/ijms22073514] of our group.

#### Protein expression analysis of differentiated PDGFRα-positive cells using western blotting

The validation of the cryopreserved differentiated PDGFRα-positive cells was done by analysing the Protein expression of Stem Cell Differentiation (SCD) markers (ALP, AGG, CD44, and FSP-1) in fibroblasts, iMSCs, and differentiated PDGFRα-positive cells. The details of the results obtained from western blot analysis can be obtained from published data [1] [10.3390/ijms22073514] (Additional file 1).

#### Morphological examination of the differentiated PDGFRα-positive cells post cryopreservation

The conservation in morphology of the cryopreserved differentiated PDGFRα-positive cells was studied using Scanning Electron Microscopy (SEM). The images of the morphological examination can be obtained from published data [1] [10.3390/ijms22073514].

## Conclusion

The main advantages of developing the present protocol were reducing the time taken to differentiate PDGFRα-positive cells, making the cells readily available for functional studies, reducing the cost of reagents, and achieving an excellent reproducibility. MTT viability assay has revealed a non-significant difference in cell survival before and after freezing, indicating no adverse effects of long-term storage on the cells, which are comparable to the freshly differentiated cells. Validation experiments using flow cytometry have shown that the efficiency of differentiation is due to increased expression of the CD140α cell surface marker, also known as PDGFRα. Gene and protein expression analysis has also highlighted the intact functional capacity of these cells to be used for further experimentation. Comparing the outcomes with relevant positive and negative control cells cultured simultaneously further provides proof of maintained differentiation. However, future studies, including cell population analysis, are warranted to reveal important information related to the ratio of differentiated vs. non-differentiated cells in this homogenous population.

## Limitations

One of the limitations of this study is obtaining high percentage of reproducibility between each round of cryopreservation and thawing. Therefore, each step of the differentiation process and thawing is critical. Secondly, although DMSO is considered as the best cryoprotectant for cell culture, its toxicity to the cells remains a concern. Hence, using optimum concentrations of components in the freezing medium containing DMSO is imperative and critical.

### Supplementary Information

**Additional file 1:** Western Blot Images.

## Data Availability

All data presented from published data have been subjected to prior approval from the concerned authors.

## Abbreviations

AGG: Aggrecan
ALP: Alkaline phosphatase
COL I: Collagen type I
CTGF: Connective tissue growth factor
DEC: Decorin
DM: Diabetes mellitus
ECM: Extracellular matrix
EDTA: Ethylenediaminetetraacetic acid
ELA: Elastin
PDGFRα-positive cells: Platelet-derived growth factor receptor-α-positive cells
iMSCs: Telomerase transformed mesenchymal stromal cells
LAA: L-Ascorbic acid
iMSCs: Telomerase transformed mesenchymal stromal cells
TIMP1: Tissue inhibitor of metalloproteinases 1
HAS3: Hyaluronic acid synthase 3
