# Supplementary material for: Cryopreservation and validation of differentiated PDGFRα-positive cells for long term usage in experimentation
Source: BMC Res Notes. 2023 Oct 19;16:280. doi: 10.1186/s13104-023-06549-y (PMC10585902; doi:10.1186/s13104-023-06549-y)
Supplement: Supplementary file 1 — Additional file 1: Western Blot Images. [file 13104_2023_6549_MOESM1_ESM.pdf]

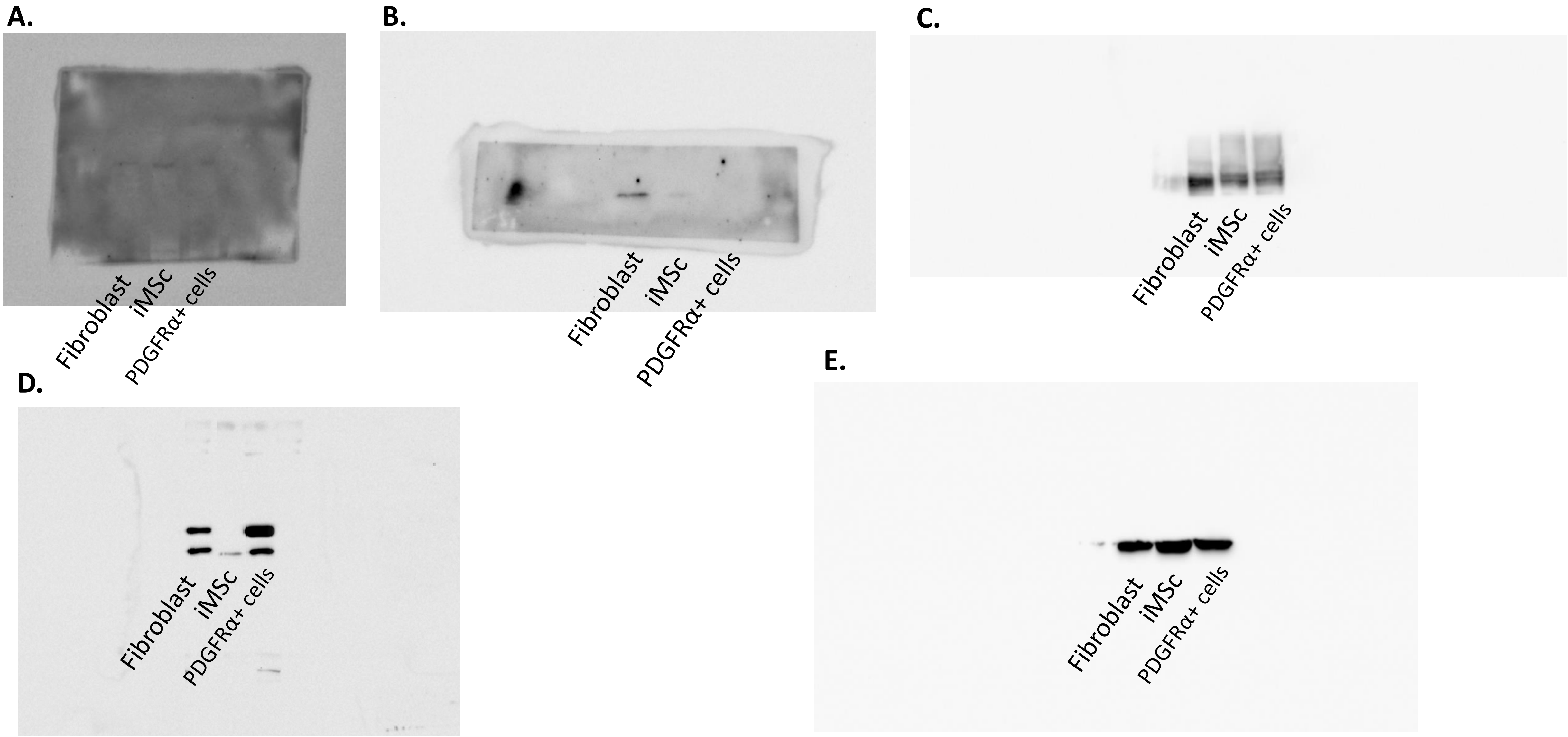

Figure S1: shows the corresponding full length western blot images from the original figure (Figure 5A) of published manuscript (Mussa et. al. 2021) . Expression was determined for the following stem cell differentiation markers: (A) AGG, (B) FSP-1, (C) CD44, (D) ALP, and (E) β-actin (loading control) in differentiated PDGFRα-positive (PDGFRα+) cells and compared with the positive control (Fibroblast) and negative control (iMSc).

Reference: Mussa, B. M., Khan, A. A., Srivastava, A., & Abdallah, S. H. (2021). Differentiated PDGFRα-Positive Cells: A Novel In-Vitro Model for Functional Studies of Neuronal Nitric Oxide Synthase. International Journal of Molecular Sciences, 22(7), 3514. MDPI AG. Retrieved from http://dx.doi.org/10.3390/ijms22073514
